# Ectopic paraesophageal mediastinal parathyroid adenoma, a rare cause of acute pancreatitis

**DOI:** 10.1186/1477-7819-2-41

**Published:** 2004-11-30

**Authors:** Christophoros N Foroulis, Sotirios Rousogiannis, Christos Lioupis, Dimitrios Koutarelos, Georgia Kassi, Athanassios Lioupis

**Affiliations:** 1 Larissa University Hospital, Department of Cardio-thoracic Surgery, 41110 Larissa, Greece; 2 Volos General Hospital, Department of General Surgery, 38221 Volos, Greece; 3 Volos General Hospital, 1^st ^Department of Internal Medicine, 38221 Volos, Greece

## Abstract

**Background:**

The manifestation of primary hyperparathyroidism with acute pancreatitis is a rare event. Ectopic paraesophageal parathyroid adenomas account for about 5%–10% of primary hyperparathyroidism and surgical resection results in cure of the disease.

**Case presentation:**

A 71-year-old woman was presented with acute pancreatitis and hypercalcaemia. During the investigation of hypercalcemia, a paraesophageal ectopic parathyroid mass was detected by computerized tomography (CT) scan and ^99m^Tc sestamibi scintigraphy. The tumor was resected via a cervical collar incision and calcium and parathormone tumor levels returned to normal within 48 hours.

**Conclusions:**

Acute pancreatitis associated with hypercalcaemia should pose the suspicion of primary hyperparathyroidism. Accurate preoperative localization of an ectopic parathyroid adenoma, by using the combination of ^99m^Tc sestamibi scintigraphy and CT scan of the neck and chest allows successful surgical treatment.

## Background

Acute pancreatitis occurring secondary to hypercalcemia is rare. Most of the time adenomas are located in the neck. However, in 10–20% cases the parathyroid adenomas are found to be located within the mediastinum [1,2]. The lower parathyroid glands develop from the third pharyngeal pouch in close association with the thymus and they may migrate along with the thymus during development. As a result they may be found commonly within the anterosuperior mediastinum. On the other hand, the superior parathyroid glands are not associated with the thymus and may even be located in the posterior mediastinum [1-3]. Paraesophageal or retroesophageal parathyroid tumors arise from superior parathyroid glands, have normal blood supply from a branch of the inferior thyroid artery and are not embryologically considered ectopic [1-3].

We present a case of paraesophageal parathyroid adenoma clinically presenting as acute pancreatitis and successfully managed surgically by the collaboration of thoracic and general surgeons, via a cervical incision.

## Case presentation

**Figure 1:**
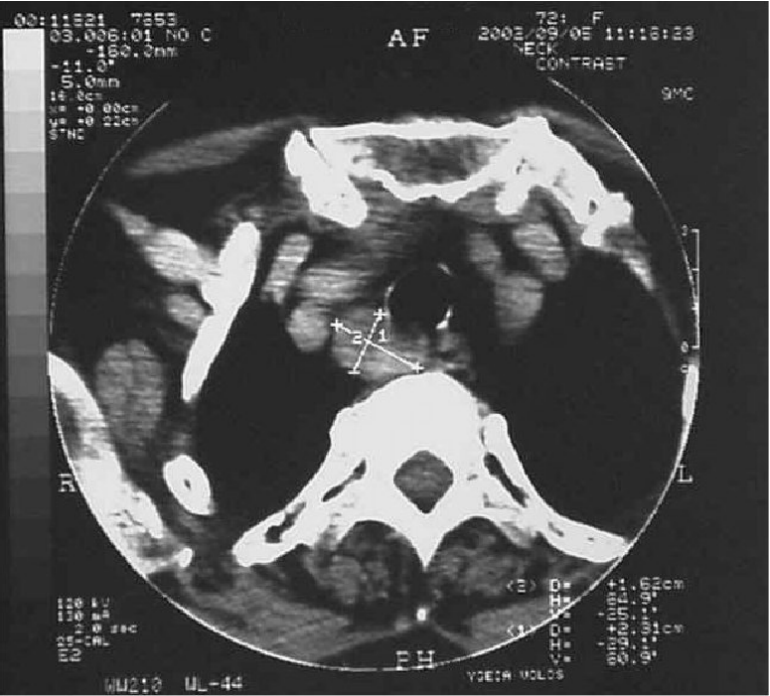
CT scan showing a paraesophageal, retrotracheal mass, close to the thoracic inlet

**Figure 2:**
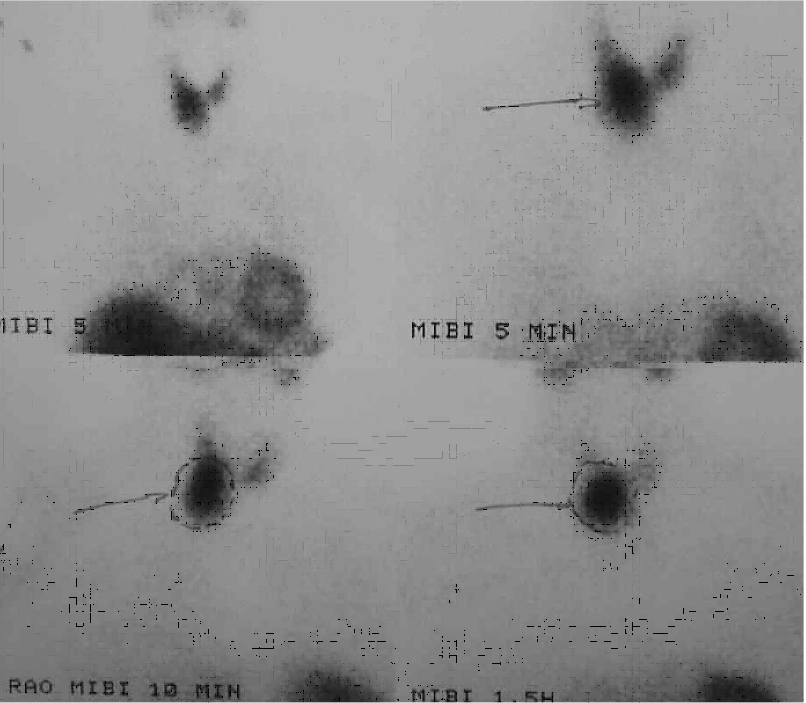
Imaging of the parathyroid paraesophageal adenoma by Tc-99m scintigraphy

A 71-year-old female was admitted with epigastric pain and vomiting lasting for more than 12 hours. She had a history of arterial hypertension and cholecystectomy two years previously. On examination she had tenderness of the upper abdomen. Blood tests showed leucocytosis (14,500 / mm^3^), increased serum levels of amylase (1,100 IU/L), LDH (550 IU/L) and calcium (14.8 mg/dl). On ultrasonography of the upper abdomen a non homogeneous appearance of the head of the pancreas was noted with a common bile duct diameter of 8.5 mm. CT scan of the abdomen confirmed the diagnosis of acute exudative pancreatitis. Acute pancreatitis subsided within 72 hours after conservative treatment. Further laboratory investigation of the hypercalcaemia revealed increased 24-hours urine calcium (465 mg), decreased serum phosphorus levels at 1.3 mg/dl, increased serum parathyroid hormone levels (771 pg/ml), normal levels of serum free T3 (FT3), free T4 (FT4), thyroid stimulating hormone (TSH), calcitonine, carcino embryonic antigen (CEA), carcinoma antigen (CA) 15-9, CA 125, alpha feto protein (AFP). Ultrasonography of the thyroid gland and the neck showed a suspicious prevertebral mass. CT scan of the thorax and neck detected a paraesophageal mediastinal mass close to the thoracic inlet. (Figure 1) ^99m^Tc sestamibi scintigraphy confirmed the diagnosis of parathyroid adenoma. (Figure 2).

**Figure 3:**
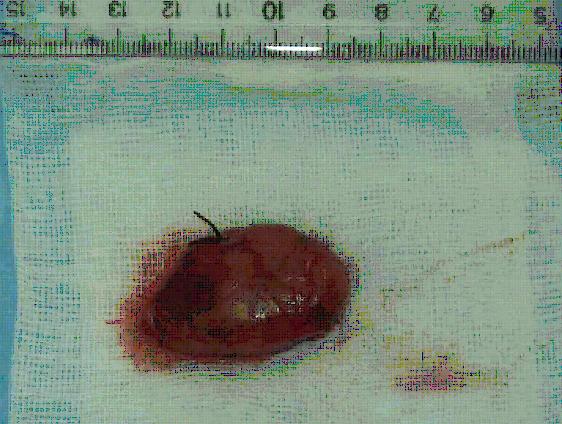
The parathyroid adenoma after resection, measuring 40 mm in its maximal dimension. The silk suture ligating the feeding vessels is shown.

After the complete subsidence of acute pancreatitis and the return of calcium serum levels at values less than 12 g/dl by hydration, the patient underwent endoscopic retrograde cholangiopancreaticography (ERCP) which failed to show bile duct lithiasis. With a diagnosis of parathyroid adenoma, resection of the parathyroid adenoma via a collar cervical incision was carried out. The tumor was easily separated from the surrounded structures (vertebra, trachea, and esophagus) by blunt dissection and the feeding vessels were found and ligated anteriorly. (Figure 3) The patient had a 6 days uneventful hospital stay. Calcium and parathyroid hormone serum levels returned to normal within 48 hours from the end of operation. A slight serum hypocalcaemia was observed over the following days and the patient received oral therapy with calcium and vitamin D to restore serum calcium levels within normal range values.

## Discussion

Recurrent episodes of acute pancreatitis secondary to hypercalcemia are an uncommon presentation of primary hyperparathyroidism [4-7]. Acute pancreatitis is reported to be associated with primary hyperparathyroidism in 1% – 8% of cases in some large published series [4-7]. Sporadically reported cases of acute pancreatitis induced by primary hyperparathyroidism, in both the recent and past medical literature, suggest that the relationship between the two clinical conditions is not incidental [8-15]. Carnaille *et al *found significantly elevated serum calcium levels to be of major importance in the development of pancreatitis in patients with primary hyperparathyroidism [7].

Increased levels of serum calcium at the first episode of acute pancreatitis should pose the suspicion of primary hyperparathyroidism. In patients with a history of cholecystectomy (as in the presented case), where the main cause of an episode of acute pancreatitis is bile duct lithiasis, the diagnosis could be missed if serum calcium levels ranged within normal values. The main causes of primary hyperparathyroidism are single or double parathyroid adenoma (80%), hyperplasia of all four or more existing parathyroid glands (15–20%) and rarely cancer of the parathyroid gland (2%) [1,2,16].

Richards *et al *reported 5%–10% of the parathyroid glands to be located in the posterior mediastinum, 20% are found substernally within the thymic tissue in the anterior mediastinum (1–2%) while 1% of the glands are located in the carotid sheath and 5% into the thyroid gland. [2] Other rare sites of ectopic parathyroid tissue are the vagus nerve sheath, the thyrothymic ligament and the pericardium [3]. By reviewing 112 patients who underwent re-operation for primary hyperparathyroidism, Wang found 39% of missing adenomas to be located in the retrotracheal space [17]. Parathyroid glands, are now found with increasing frequency in the visceral compartment of the mediastinum (aortopulmonary window and right pulmonary artery, close to the tracheal bifurcation), because of the improvement of the imaging techniques (^99m^Tc sestamibi scintigraphy). The frequency of this occurrence at the moment is uncertain [2].

Many investigators advocate the need for the concordance of at least two diagnostic modalities before surgical excision. The combination of ^99m^Tc sestamibi scintigraphy and CT scan of the chest and neck gives important information to proceed with surgery and to minimize the risk of re-operation for recurrent hyperparathyroidism in the future [3,16,18,19]. The combination of both techniques had 100% sensitivity and 97.4% positive predictive value for the detection of the cause of primary hyperparathyroidism [18]. The spectrum of diseases demonstrated with ^99m^Tc scintigraphy includes eutopic parathyroid disease, ectopic parathyroid disease, solitary, double or multiple parathyroid adenoma, cystic adenoma, lipoadenoma, multiple endocrine neoplasia, entities with atypical washout and non-parathyroid entities that take up ^99m^Tc sestamibi (normal and pathologic cervical, supraclavicular, axillary lymph nodes, hyperplastic thymus, focal soft tissue uptake from a sarcoid or carcinoid tumor) [3]. The addition of early lateral views to the conventional ^99m^Tc sestamibi scintigraphy gives more information to the surgeon, concerning the depth of the lesion in atypical sites [20].

CT scan with intravenously injected contrast material has a low overall sensitivity of 45%–55% in primary hyperparathyroidism, but it is helpful mainly in the detection of ectopic mediastinal parathyroid adenomas [2,3]. Magnetic resonance imaging (MRI) of the neck and chest has a sensitivity of about 80%. The sensitivity of MRI is higher for the detection of ectopic mediastinal parathyroid adenomas (88%) [3]. Selective angiography combined with venous parathyroid hormone sampling has sensitivity between 60% and 85%. However, selective angiography is an aggressive and complicated approach and it is not advised as the initial approach in primary hyperaparathyroidism [2,3]. Single photon-emission computed tomographic (SPECT) sestamibi scintigraphy of the neck and thorax has the capability of three-dimensional assessment and it is considered to be the optimal method for the evaluation of parathyroid disease, especially that of mediastinum for ectopic parathyroid glands [21-24]. Fusion of sestamibi SPECT images onto the CT images using a software package, as described by Patrick et al, gives excellent information on the exact localization of ectopic parathyroid tissue [19]. FDG-PET was found to have higher sensitivity than the sestamibi-SPECT in a prospective study by Neumann *et al *for preoperative detection and localization of parathyroid adenomas; high cost and limited availability of the scanners restrict its use as first-line examination in primary hyperparathyroidism [25].

Paraesophageal mediastinal adenomas are resected via a cervical incision in the majority of cases [1,2,16,26]. By retracting the thyroid gland and trachea to the opposite side, a finger can be inserted into the pretracheal space, even down to the mediastinum, to palpate the tumor. If the tumor is localized by finger palpation, it is easy to mobilize by blunt (finger) dissection and to expose it into the operating field. The vascular pedicle is the only structure that needs to be ligated. When an ectopic cervical or paraesophageal parathyroid adenoma is detected preoperatively by imaging studies, intraoperative frozen section of the adenoma and of a homolateral parathyroid gland, on which normal parathyroid tissue will be confirmed, precludes diffuse parathyroid hyperplasia. A targeted operation can then be chosen, which has the advantage of minimizing the time of operation and avoiding serious hypocalcemia in the immediate postoperative period. [26]

## Conclusions

An ectopic paraesophageal parathyroid adenoma may be manifested with an episode of acute pancreatitis. Preoperative investigation for exact localization of an adenoma should include two imaging studies, preferably Tc-99m sestamibi scintigraphy or sestamibi-SPECT scintigraphy of the neck and chest and CT scan of the neck and chest. Resection of an ectopic paraesophageal adenoma is easily accomplished via a cervical incision and blunt mobilization of the tumor.

## Competing interests

The author(s) declare that they have no competing interests.

## Authors contributions

**CNF **has made contribution to the conception, design and drafting of the article, was involved in the critical revision of the article.

**S R **has made contribution to the conception, design and drafting of the article.

**C L **has made contribution to the conception and design of the article.

**D K **has made contribution to the conception and design of the article.

**G K **has made contribution to the conception and design of the article, was involved in the critical revision of the article.

**AL **has made contribution to the conception and design of the article, was involved in the critical revision of the article.

All the authors have read and approved the final version of the manuscript.
